# Oxidative Stress, Mitochondrial Dysfunction, and Neuroprotection of Polyphenols with Respect to Resveratrol in Parkinson’s Disease

**DOI:** 10.3390/biomedicines9080918

**Published:** 2021-07-30

**Authors:** Heng-Chung Kung, Kai-Jung Lin, Chia-Te Kung, Tsu-Kung Lin

**Affiliations:** 1 Center for Mitochondrial Research and Medicine, Kaohsiung Chang Gung Memorial Hospital and Chang Gung University College of Medicine, Kaohsiung 83301, Taiwan; rexkun18@gmail.com (H.-C.K.); kj30728@gmail.com (K.-J.L.); 2 Department of Biology, Krieger School of Arts and Sciences, Johns Hopkins University, Baltimore, MD 21218, USA; 3 Department of Family Medicine, National Taiwan University Hospital, Taipei 100225, Taiwan; 4 Department of Emergency Medicine, Kaohsiung Chang Gung Memorial Hospital and Chang Gung University College of Medicine, Kaohsiung 83301, Taiwan; 5 Department of Neurology, Kaohsiung Chang Gung Memorial Hospital and Chang Gung University College of Medicine, Kaohsiung 83301, Taiwan; 6 Center of Parkinson’s Disease, Kaohsiung Chang Gung Memorial Hospital and Chang Gung University College of Medicine, Kaohsiung 83301, Taiwan

**Keywords:** Parkinson’s disease, mitochondria, polyphenol, resveratrol, neuroprotection, oxidative stress, antioxidant, autophagy, clinical trials, aging

## Abstract

Parkinson’s disease (PD) is the second most common neurodegenerative disease and is characterized by dopaminergic neuronal loss. The exact pathogenesis of PD is complex and not yet completely understood, but research has established the critical role mitochondrial dysfunction plays in the development of PD. As the main producer of cytosolic reactive oxygen species (ROS), mitochondria are particularly susceptible to oxidative stress once an imbalance between ROS generation and the organelle’s antioxidative system occurs. An overabundance of ROS in the mitochondria can lead to mitochondrial dysfunction and further vicious cycles. Once enough damage accumulates, the cell may undergo mitochondria-dependent apoptosis or necrosis, resulting in the neuronal loss of PD. Polyphenols are a group of natural compounds that have been shown to offer protection against various diseases, including PD. Among these, the plant-derived polyphenol, resveratrol, exhibits neuroprotective effects through its antioxidative capabilities and provides mitochondria protection. Resveratrol also modulates crucial genes involved in antioxidative enzymes regulation, mitochondrial dynamics, and cellular survival. Additionally, resveratrol offers neuroprotective effects by upregulating mitophagy through multiple pathways, including *SIRT-1* and AMPK/*ERK* pathways. This compound may provide potential neuroprotective effects, and more clinical research is needed to establish the efficacy of resveratrol in clinical settings.

## 1. Introduction

Parkinson’s disease (PD) is the second most common neurodegenerative disease after Alzheimer’s disease (AD) [1]. Age is the biggest risk factor of PD, affecting more than 1% of the population over the age of 60 [2]. PD was first described as a neurological syndrome by James Parkinson in 1817, who also observed its clinical manifestations, such as rigidity, bradykinesia, gait disturbance, and a resting tremor [3]. A century later, in 1912, Fritz Heinrich Lewy discovered the first major pathological hallmark of PD—neuronal inclusions—in the brain of PD patients [4]. These neuronal inclusions, termed Lewy bodies, would later be discovered to predominantly contain α-synuclein protein aggregation [5]. It is well accepted that PD is a dopamine-deficiency disorder with L-3,4-dihydroxyphenylalanine (L-DOPA)—a precursor for dopamine—being the gold standard for the symptomatic treatment of this disease since the 1970s [6]. However, dopaminergic therapy only serves as a symptomatic treatment for PD, and a cure for PD has yet to be discovered [7]. 

Progressive dopaminergic neuronal loss has been identified as another pathological hallmark of PD. According to Braak’s six-stage scheme, neuronal involvement in the early stages is typically confined to the medulla oblongata, but gradually spreads to other parts of the brain as the disease progresses [8]. However, more recent research has challenged the generality of Braak’s scheme, as cases with different progression patterns were reported.

Though the primary cause of PD remains unknown, researchers have uncovered more than 23 hereditary familial gene mutations related to PD [9,10]. Even though only several mutations have been identified as disease-causing or disease-related in a small percentage of PD cases, findings generated from this genetic research have resulted in further clarification of PD’s pathophysiology [10]. For example, identifying point mutations in the SNCA gene encoding the pathogenic protein α-synuclein in PD patients has helped researchers identify protein misfolding and/or overexpression as possible mechanisms for Lewy body pathology [11]. Mutations in the parkin gene, which codes for a ubiquitin E3 protein ligase, have been identified in young-onset recessive familial PD. This demonstrates the involvement of damaged mitochondrial degradation via the autophagy–lysosomal pathway and ubiquitin–proteasome clearance dysfunction in PD pathogenesis [12]. Other possible mechanisms leading to PD pathology include oxidative damage, mitochondrial dysfunction, the accumulation of α-synuclein, calcium (Ca^2+^) imbalance, the disruption of endo-lysosomal function and autophagy, and neuroinflammation [13,14]. 

Among these, oxidative damage and the consequential mitochondrial dysfunction remains on the central stage of PD pathogenesis. Given the high-energy usage of dopaminergic neurons, mitochondria, the cell’s main producer of adenosine triphosphate (ATP), have been proposed to play a role in the development of PD [15,16,17]. Mitochondria, as the major generator of cellular energy ATP through the process of oxidative phosphorylation (OXPHOS), are also an important source of reactive oxygen species (ROS), as electrons constantly leak through the electron transport chain (ETC) [18]. Usually mitochondrial anti-oxidative systems detoxify ROS, maintaining a balance between harmful radical production and antioxidative protection [19]. Once an imbalance occurs, oxidative stress rises and macromolecules in the mitochondrial structure are susceptible to oxidative damage [20]. As damaged macromolecules resulting from oxidative stress accumulate in the mitochondria, the organelle’s function is disrupted [21]. Eventually, this leads to the release of cytochrome c from the mitochondria and the triggering of cell apoptosis, which can be observed in the dopaminergic neuronal death of PD [21].

The involvement of oxidative stress in PD is also supported by epidemiology studies. As of 2016, there were around 6.1 million people with PD, which was up from 2.5 million in 1990 [22]. Though the burden of PD more than doubled over the 26 years, with an increasing number of older people and longer life expectancies, this rise cannot be solely attributed to an aging population, as the age-standardized prevalence rate rose by 27.1% as opposed to the 74.3% increase in the crude prevalence rate [22]. In addition to genetic predispositions, other non-genetic environmental risk factors of PD, including exposure to chemicals such as pesticides, herbicides, and heavy metals, have been proposed by epidemiological and toxic experiments [23,24,25]. 

Thus, in this review article, we will first explore the basics of mitochondrial biology, including its structure, function, and maintenance mechanism. We will place emphasis on how oxidative stress causes mitochondrial dysfunction and the roles both oxidative stress and mitochondrial dysfunction play in PD pathophysiology. Autophagy, its mechanism and ability to maintain mitochondrial health/homeostasis, will be briefly introduced. Usage of the antioxidative compound resveratrol, its possible mechanisms in reducing neuronal damage, and data on clinical trials will be discussed.

## 2. Mitochondrial Biology 

Mitochondria are organelles critical to the cell viability as the major producer of ATP required for the cell’s functioning. This double-membraned organelle is composed of an outer membrane (OM) and an inner membrane (IM) separated by the intermembrane space (IMS) [26]. The cristae, convoluted IM folds, enclose the mitochondrial matrix, which holds enzymes for metabolic reactions and a genome separate from that of the nucleus [27]. Catabolic processes occur within the mitochondria, where molecules such as nucleotides, pyruvate derived from glucose or lactate, heme, and fatty acids, are broken down and oxidized to provide electrons for generating chemical energy in the form of ATP [28,29,30]. Electrons are passed to cofactors nicotinamide adenine dinucleotide (NADH) and flavin adenine dinucleotide (FADH_2_) in the tricarboxylic acid (TCA) cycle [29]. 

**Figure 1:**
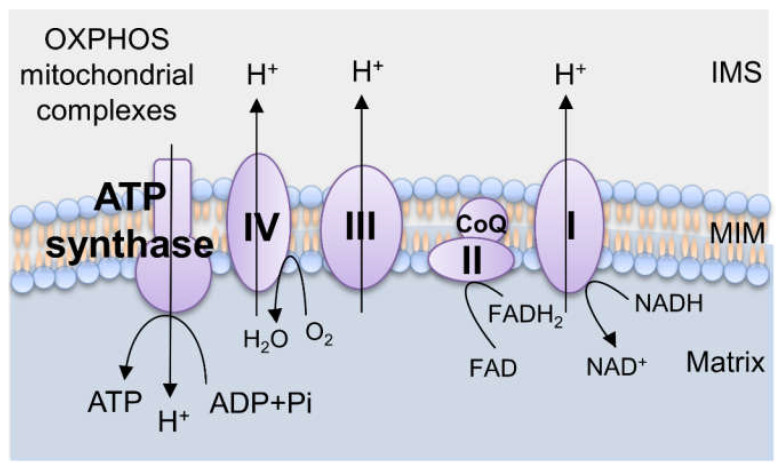
A schematic illustration of the OXPHOS system. There are five main protein complexes involved in OXPHOS: the mitochondrial complexes I-IV and F0F1 ATPase. As electrons are transported through the mitochondrial complexes (I-IV) in a series of redox reactions, energy is transferred to transport protons across the mitochondrial IM, creating an electrochemical potential to drive protons back to the mitochondrial matrix through the F0F1 ATPase and transform ADP to ATP (phosphorylation). OXPHOS, oxidative phosphorylation; IMS, intermembrane space; IM, inner membrane.

Once the electrons are passed onto the ETC from the reduced electron carriers, they are shuttled down the different subunits of complexes I–IV embedded on the IM. As electrons are shuttled down the ETC, complexes I, III, and IV pump hydrogen from the matrix across the IM into the IMS in order to generate an electrochemical gradient across the IM, known as the mitochondrial membrane potential (ΔΨm) [31,32]. Complex V (F_0_F_1_ ATPase), driven by the proton gradient generated from the series of oxidation and reduction, serves as a rotary molecular motor that phosphorylates ADP into ATP [33,34,35]. Thus, it completes the process of synthesizing chemical energy in the form of ATP by consuming nutrients and oxygen by OXPHOS (Figure 1). 

Though the composition of mitochondria is plastic and varies across different species and cells, most human mitochondrial proteome is made up of around 1000 to 1500 proteins, 99% of which are encoded by nuclear DNA, with only various small portions of mitochondrial proteins encoded by the unique mitochondrial DNA (mtDNA) [28,36,37]. The 16569 base pair, double-stranded, circular mtDNA within the matrix consists of 37 genes and codes for 13 polypeptides critical to the OXPHOS complexes, and a full set of protein translation machinery, including 22 mitochondrial tRNA, a 16S rRNA (large ribosomal unit), and a 12S rRNA (small ribosomal unit) [38,39]. Each human mitochondrion typically contains 2–10 copies of mtDNA and up to 1000 copies per cell, but the actual number can vary according to the cell type [40,41].

### 2.1. Mitochondria and ROS Generation

Although mitochondria are highly efficient organelles for energy production, there is a constant leak of electrons from the chain as they flow down the ETC, particularly from Complex I (NADH coenzyme Q reductase) and Complex III (ubiquinol cytochrome c reductase) [42]. Being the largest consumer of oxygen in the cell, the mitochondrion generates almost 90% of all ROS in the body as a metabolic byproduct under physiological conditions [18,43,44]. Approximately 0.2–2% of electrons that flow down in the ETC leak out under normal physiological conditions, as opposed to following the usual transfer order [45]. In the presence of oxygen molecules (O_2_), these leaked electrons are taken up by O_2_ on site and form the superoxide anion radical O_2_^•−^ (primary ROS) [46]. Usually, O_2_^•−^ is readily dismutated by manganese (Mn)-superoxide dismutase (Mn-SOD/SOD2) in the mitochondrial matrix and copper/zinc (Cu/Zn)-SOD (SOD1) in the cytoplasm and IMS, releasing hydrogen peroxide (H_2_O_2_). H_2_O_2_ is typically more stable and can be converted into H_2_O by additional enzyme processes [47]. However, in the presence of transition metals, such as iron or copper (Cu), H_2_O_2_ can react with O_2_^•−^ to form the harmful hydroxy radical (HO^•^) via the Haber–Weiss reaction [48,49]. In Complex I, electrons are passed down from NADH to flavin mononucleotide (FMN), then to seven iron–sulfur centers, and then finally to co-enzyme Q (CoQ) [50]. O_2_^•−^ can be formed inside the matrix when the reduced form of FMN interacts with oxygen molecules, a reaction favored when the NADH/NAD^+^ ratio is high in the matrix [50]. As opposed to Complex I, Complex III, responsible for the transfer of electrons from ubiquinol to cytochrome c in a process called a Q-cycle, produces less ROS [32,50,51,52]. ROS is produced when ubisemiquinone (QH^−^) of the Q_o_ site leaks the electron to O_2_ [32]. Other than the ETC, many other sites within the mitochondria (the mitochondrial matrix, IMS, and OM) may also produce O_2_^•−^ or H_2_O_2_. Thus, as Murphy et al., have reviewed, it is convenient to divide most of these into sites that interact with the matrix NADH pool and those that are connected to the CoQ pool within the inner membrane [18]. 

### 2.2. Mitochondrial Oxidative Stress and Antioxidative Systems 

In order to manage the oxidative stress and preserve cellular homeostasis, mitochondria have their own intricate antioxidative system consisting of multiple signaling molecules and enzymes. The main antioxidative defense system in the cell is superoxide dismutases (SODs), which catalyze the dismutation of O_2_^•−^ into H_2_O_2_ and O_2_ with the help of cofactors, such as Cu, Zn, and Mn [53]. Three isoforms of SODs exist—cytoplasmic Cu/Zn-SOD (SOD1), mitochondrial Mn-SOD (SOD2), and extracellular Cu/Zn-SOD (SOD3)—with SOD1 and SOD2 more associated with oxidative stress given their intracellular localization [54]. SOD1 is predominantly located in the cytoplasm, but it is also present in the IMS, where it provides antioxidative properties [55].

Once H_2_O_2_ is formed from the dismutation of O_2_^•−^, it can be eliminated by catalase, which have been found in liver and cardiac mitochondria [56,57]. Furthermore, mitochondria utilize two other pathways that require the reductive ability of NADPH to degrade H_2_O_2_: the glutathione (GSH) and thioredoxin (TRX) systems [58]. In addition to being an enzyme cofactor, the tripeptide GSH carries an active thiol group and acts as an antioxidant by directly interacting with ROS/RNS and electrophiles [59]. Two molecules of GSH are oxidized into glutathione disulfide (GSSG) in order to eliminate H_2_O_2_ by GSH peroxidase (GPX) isozymes (GPX1 and GPX2) in the mitochondria [58,60]. Additionally, GSH can be directly oxidized by radicals such as HO^•^, forming thiyl radicals, which fuse together to produce GSSG [59,60]. GSH levels are restored through the reduction of GSSG in the presence of NAPDH, a reaction catalyzed by glutathione reductase (GR) [61]. In the TRX system, peroxiredoxin (PRX), particularly isoenzymes PRX3 and PRX5 in the mitochondrial matrix, clears H_2_O_2_ with a peroxidatic cysteine (CysP) in its active site [62,63]. After the sequestration of H_2_O_2_, PRX3 and PRX5 are reactivated by the reductive power of TRX2, which is then reactivated in the presence of NADPH by thioredoxin reductase-2 (Trx2) [58]. 

### 2.3. Oxidative Stress and Mitochondrial Dysfunction

Before we discuss the harmful nature of ROS, we must first recognize their role in normal physiological function. Recent findings have begun to show that even though ROS can cause oxidative damage, they are critical to different signaling pathways as stress-responsive mediators [51,64,65]. For example, ROS such as H_2_O_2_ participate in the activation of the nuclear factor kappa-light-chain-enhancer of the activated B cells (NF-κB) signaling pathway, which is paramount in the regulation of both inflammation and the immune system [66,67]. Schmidt et al., found that overexpression of catalase, which converts H_2_O_2_ into H_2_O, led to the inhibition of NF-κB activation [68]. On the other hand, overexpression of SOD1, which catalyzes O_2_^•−^ into H_2_O_2_, upregulated NF-κB activation [68]. Furthermore, ROS can modulate gene expression. Under oxidative conditions, ROS helps to activate the expression of nuclear transcription factor-erythroid 2-related factor 2 (Nrf2), which regulates antioxidative responses and cytoprotective effects [69]. Consequently, it is not the mere presence of ROS that is harmful to the body, but an overabundance of ROS resulting from a disruption of the balance between ROS generation and elimination. 

Thus, once the balance is tipped towards the generation of ROS, and accumulation occurs, there is harmful oxidative stress. Being highly reactive, ROS will interact with macromolecules, such as nucleic acids, proteins, and lipids, damaging these molecules and cellular organelles [70]. mtDNA is particularly susceptible to oxidative damage, being so close to the site of ROS production and having a less effective DNA polymerase repair system [71]. Consequently, it is not uncommon for people to have mtDNA mutations: 1 to 200 people have one or more of the ten most common mtDNA mutations [72]. However, not all of these mtDNA mutations will manifest into diseases, as normal mtDNA can coexist with mutated mtDNA in a phenomenon known as heteroplasmy [72,73]. All heteroplasmic mtDNA mutations but one are considered recessive, and an extremely large number of mutations would be required for the alterations to manifest into a phenotypic disease [74]. However, once enough mtDNA mutations accumulate, they can give rise to faulty proteins or result in reduced expression of critical proteins in the ETC [75]. The lack of functional proteins in the ETC, particularly in Complexes I and III, can decrease ATP production and increase the reduction of O_2_ into ROS, creating a vicious cycle that ultimately leads to complete organelle dysfunction [76]. 

Furthermore, since OXPHOS and ROS production occur on the IM, the IM is particularly susceptible to lipid peroxidation [77]. Normally, the IM is only permeable to neutral molecules, such as carbon dioxide, water, and oxygen; the permeability of charged particles, such as protons, is limited in order to establish and maintain the ΔΨm that drives ATP synthesis [78]. However, lipid peroxidation can increase the IM’s proton permeability and change fluidity and other biophysical properties of the IM, consequently reducing the efficiency of OXPHOS [79,80]. Mitochondrial permeability transition (MPT), the process of non-selective permeabilization of the inner membrane, is partially driven by oxidative stress [81,82,83]. Studies have shown that oxidation of NADPH, with its role in antioxidative protection, further drives MPT, as NADPH can reduce GSH and thioredoxin (TSH), which participate in the removal of H_2_O_2_ by mitochondrial GPX and thioredoxin peroxidase (TPX), respectively [81,84]. Another major factor in the regulation of MPT is the oxidation state of mitochondrial thiols: thiol oxidants, such as diamide and 4,4P-diisothiocyanato-stilbene-2,2P-disulfonic, promote MPT [85,86], while dithiothreitol and other thiol reductants inhibit MPT [81,87,88]. The formation of mitochondrial permeability transition pores (mPTP) during MPT can increase IM permeability to 1.5 kDa and eventually lead to cell death [89]. Furthermore, ROS can also alter the structure and functions of transporters and enzymes involved in OXPHOS [90]. 

ROS damage to proteins, nucleic acids, and lipids can further disrupt Ca^2+^ homeostasis [79]. Ca^2+^ plays a huge role in regulating the functioning of the cell; Ca^2+^ is critical in multiple intracellular signaling pathways, including muscle contractions, cell differentiation, neuro/enzyme secretion, cell proliferation, and cell death [91]. In addition to the endoplasmic reticulum (ER), mitochondria also serve as a storage for Ca^2+^, and an appropriate Ca^2+^ concentration in the mitochondria is necessary for proper mitochondrial function [92]. Metabolic processes, such as the activation of mitochondrial dehydrogenases, will not be stimulated when the Ca^2+^ concentration is too low, and an extremely high Ca^2+^ concentration will trigger cell apoptosis or necrosis [93,94]. In contrast to the OM, which is highly permeable to Ca^2+^, the IM is much less permeable to Ca^2+^ and determines the rate at which Ca^2+^ enters the mitochondrial matrix [95]. Ca^2+^ influx through the IM is regulated by a highly specific ion channel with a mitochondrial calcium uniporter (MCU) as the ion-conducting pore, and is driven by ΔΨm [96,97]. Oxidants increase the release of Ca^2+^ from the ER while downregulating the extrusion of Ca^2+^ through the plasma membrane [98]. Combined with the formation of mPTP from IM thiol oxidation, there will be an increase in uptake of Ca^2+^ by the mitochondrial matrix, partially to protect cells against cytosolic Ca^2+^ overload [92]. Interestingly, mPTP formation is sensitive to both ROS and Ca^2+^ overload, which indicates the presence of an amplification loop that triggers MPT through either Ca^2+^-induced Ca^2+^ release or ROS-induced ROS release [99]. 

### 2.4. Oxidative Stress and Cell Death

Over the past decade, the Nomenclature Committee on Cell Death (NCCD) has formulated guidelines classifying cell death according to morphological, biochemical, and functional properties with subroutines focusing on mechanistic and essential aspects. In 2018, NCCD proposed an updated set of molecularly-oriented classification for cell death, including intrinsic apoptosis, extrinsic apoptosis, mitochondrial permeability transition (MPT)-driven necrosis, necroptosis, ferroptosis, pyroptosis, parthanatos, entotic cell death, NETotic cell death, lysosome-dependent cell death, autophagy-dependent cell death, and immunogenic cell death [100]. Of these, apoptosis traditionally refers to cell death in which a very specific set of morphological features can be observed: chromatin condensation, cell shrinkage (pyknosis), protein breakdown, nuclear fragmentation (karyorrhexis), and plasma membrane blebbing activities [101]. Eventually, small intact vesicles, typically called apoptotic bodies, form and are engulfed by nearby cells with phagocytic activity [100]. Autophagy manifests with extensive cytoplasmic vacuolization, and similarly results in phagocytic uptake and degradation via lysosomal activity [100,102]. 

Finally, necrosis shows no distinctive morphological feature of type I and II cell deaths and disposes of cell corpes without obvious phagocytic and lysosomal activity [100,103].

It is critical that apoptosis occurs at an appropriate rate in order to maintain tissue homeostasis, as apoptosis clears away damaged cells [104]. Several mechanisms work in conjunction to regulate the onset of apoptosis in a cell, the majority of which are related to the mitochondria [105,106,107,108]. Although the mitochondria can serve as a Ca^2+^ reservoir and buffer, there is a limit to the amount of Ca^2+^ they can hold [109,110]. The loss of balance between the Ca^2+^ influx and efflux through the plasma membrane as a result of oxidative stress leads to a sustained increase in cytoplasmic Ca^2+^ concentration, which in turn raises the mitochondrial Ca^2+^ uptake [111]. Ca^2+^ overload in the mitochondrial matrix over an extended period of time triggers prolonged mPTP opening, which can cause mitochondrial IM permeabilization, irregular ETC function, membrane potential dissipation, termination of ATP production, organelle swelling, OM rupture, and eventually cell necrosis [112]. In addition to necrosis, rupture of the OM also triggers the release of cytochrome c into the cytoplasm, activating the apoptotic pathway [82]. 

Cardiolipin is a type of lipid only found on the IM, and is bound to cytochrome c [113]. It is hypothesized that one of cardiolipin’s acyl chains is attached to a hydrophobic pore of cytochrome c, while the other acyl chains extend into the phospholipid bilayer [114]. Research has shown that cardiolipin oxidation “breaches” the hydrophobic and electrostatic affinity between cardiolipin and cytochrome c on the IM and even promotes cytochrome c mobilization from the mitochondria [115]. Once proapoptotic factors such as cytochrome c are released from the IMS, they would still need to pass through the OM. Mitochondrial outer membrane permeabilization (MOMP), similar to IM permeabilization, most likely occurs when membrane-spanning pores allow IMS proteins to be released [116]. MOMP is “the point of no return” in the cell apoptosis pathway and is tightly regulated by several BCL-2 proteins [117]. Pro-apoptotic BH3-only proteins associate with Bcl-2-associated X protein (BAX) and (B cell lymphoma 2 homologous antagonist killer) BAK to trigger MOMP only in apoptotic cells; BAX and BAK are inactivated in non-apoptotic cells by anti-apoptotic proteins Bcl-xL or MCL-1 [118]. Once pro-apoptotic factors are released into the cytoplasm, they can trigger several apoptotic pathways. For example, cytochrome c interacts with procaspase 9, apoptotic peptidase activating factor 1 (Apaf-1), and ATP to trigger apoptosome formation, which in return activates caspase-3, 9, and 7 [119]. Consequently, oxidative stress and mitochondrial dysfunction are known to be involved with degenerative diseases such as PD, which are often characterized by a progressive loss of physiological function as a result of cumulative cell death [120].

## 3. Parkinson’s Disease, Oxidative Stress, and Mitochondrial Dysfunction 

One of the major breakthroughs in linking PD to oxidative stress and mitochondria dysfunction is the development of parkinsonism symptoms in drug abusers who took the drug 1-methyl-4-phenyl-1,2,3,6-tetrahydropyridine (MPTP) in the 1980s [121]. In addition to developing clinical/physical parkinsonism symptoms, post-mortem analysis showed significant lesions of dopaminergic neurons in the substantia nigra pars compacta (SNpc) [122]. MPTP itself is not toxic; however, being lipophilic, MPTP is able to pass through the blood–brain barrier [123]. Once within the brain, MPTP is transformed into the toxic metabolite, 1-methyl-4-phenylpyridinium, or MPP+, by monoamine oxidase (MOA) B in glial cells [121]. Interestingly, MPP+ is an extremely great substrate for the DA uptake site, so MPP+ molecules are taken up and concentrated in dopaminergic neurons, particularly in the mitochondria [124]. Once MPP+ reaches a toxic level within the mitochondria, it inhibits Complex I of the ETC, reducing ATP production and increasing ROS generation [125]. After the identification of MPTP as a cause of parkinsonism, Schapira et al., found that in patients with sporadic PD, Complex I activity was decreased in dopaminergic neurons of the SNpc [126]. Given these findings, researchers were able to establish the critical role of mitochondria, particularly Complex I inhibition, in the pathogenesis of PD. The involvement of mitochondrial dysfunction in PD pathophysiology was further strengthened by research that identified exposure to several pesticides that acted as mitochondrial toxins in PD occurrences [127,128,129]. Rotenone, another natural Complex I inhibitor extracted from plant roots, was found to induce parkinsonism in rodents [130,131]. 

With mitochondria being the source of 90% of cellular ROS and mtDNA coding for critical polypeptides in the ETC, mtDNA damage was also proposed as a possible factor in the pathogenesis of PD [132]. mtDNA mutations and deletions in the human brain occur at a low rate in young humans, but the mtDNA alteration rate greatly increases with age and is particularly high in the elderly [133]. There is a 2.6-fold increase in mtDNA mutations from age 26 to age 80 [134]. Once a certain threshold expression is reached, mitochondrial and cellular function are altered, compromising cellular homeostasis and eventually causing cell death [135]. Maintaining the integrity of mtDNA requires the help of many nuclear-encoded proteins as well, including mtDNA polymerase gamma 1 (*POLG1*), POLG1 mitochondrial transcription factor (*TFAM*) A, DNA helicase Twinkle (*TWNK*), and the single-stranded binding protein (mtSSB) [136]. Mutations in these genes have been associated with a higher risk of PD and PD symptoms, suggesting that mtDNA mutations may play a role in PD pathogenesis [136]. 

The identification of other genetic mutations in both familial and sporadic PD have also elucidated mitochondria’s role in PD. *PINK1*, which codes for PTEN-induced serine/threonine kinase 1, and *PRKN*, which codes for E3 ubiquitin ligase parkin, work together and serve as major players in mitochondrial quality control [137]. Table 1 below provides a list of genetic mutations related to PD/parkinsonism and their functional association with the mitochondria. 

## 4. Polyphenols and Their Properties

**Figure 2:**
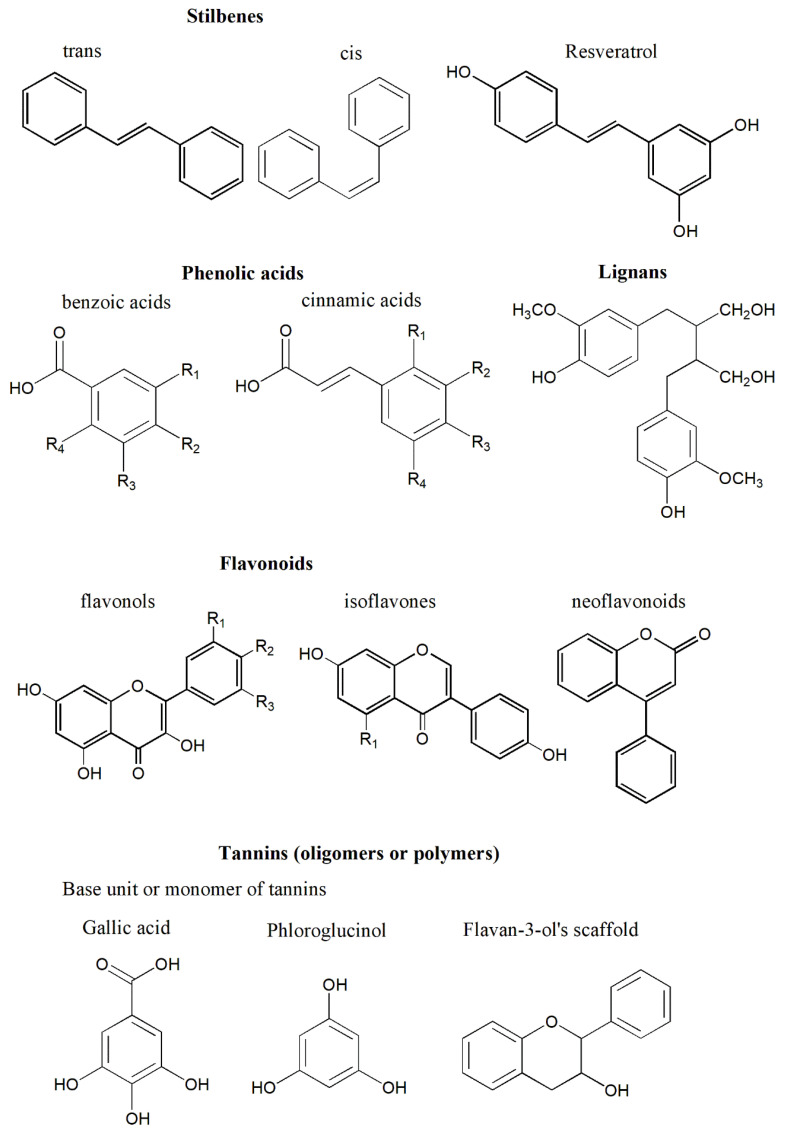
The chemical structures of polyphenols. Polyphenols are abundant phytochemicals in the human diet, with a great variety in molecular size and structure. These compounds are classified into different groups according to chemical structure, including the stilbenes (e.g., resveratrol), phenolic acids, flavonoids, tannins, and lignans.

From a chemical perspective, polyphenols are a collective group of natural compounds that contain phenolic structures [172]. Polyphenolic compounds have a wide range of structures, from those with one benzene ring to those with multiple rings [173]. Consequently, polyphenols can be classified into several main groups—including stilbenes, phenolic acids, and flavonoids, lignans, and tannins—and other smaller groups based on the compound’s structure (Figure 2) [173,174]. Polyphenols can be found naturally in tea, fruits, flowers, vegetables, and numerous other kinds of foods and plants [175]. 

Over the past few decades, animal models, human cohort, and case control studies have demonstrated that specific polyphenols possibly benefit the health status against certain diseases, including type 2 diabetes, cardiovascular diseases (CVD), and neurodegenerative diseases [176,177,178]. Several studies have found an inverse correlation between the onset of certain cancers and one’s dietary consumption of vegetables and fruits [179]. At a cellular level, polyphenols can act as chemo-preventive agents through several mechanisms [176]. These mechanisms include the regulation of gene expression and activity of certain proteins involved in cell cycle progression [180,181], elimination of carcinogenic compounds [182], and inhibition of cell proliferation through the upregulation of apoptosis pathways [183]. 

Polyphenols have also been noted to be beneficial for cardiovascular health [184]. Though the exact benefits of each polyphenol remain uncertain, drinking a moderate amount of red wine or tea, both of which are rich in polyphenols, has regularly been associated with a lower risk of CVD [185,186]. Certain polyphenols have been noted to reduce blood pressure by enhancing the formation of vasodilative nitric oxide [187], impede the oxidation of low density lipoprotein (LDL) [188], and improve endothelial function [176,189]. 

In addition to lowering the risk of certain cancers and CVD, polyphenols have been found to exhibit neuroprotective effects, thus delaying the onset of or lowering the risk of neurodegenerative diseases, such as Alzheimer’s and Parkinson’s [190,191]. Polyphenols, such as some flavanones, are able to pass through the blood–brain barrier and directly protect or stimulate neurons by shielding neurons against oxidative stress or amyloid-β neuronal damage [176,192]. 

Due to their wide range of health benefits, polyphenols may have important preventive and therapeutic uses for cancer, CVD, and other degenerative diseases in the future [193]. Paramount to these health benefits is the antioxidative effects of polyphenolic compounds [194]. A major factor in the development of the aforementioned diseases is oxidative DNA damage, which can lead to cell death or transformation [195]. Polyphenols are capable of alleviating such damage and reducing oxidative stress through three main methods: inhibiting ROS production by suppressing enzymatic activity or chelating metal ions that are able to create free radicals, scavenging ROS, and upregulating antioxidative mechanisms [196]. Studies have shown that certain polyphenols are able to suppress lipoxygenase, cyclooxygenase, NADH oxidase, and other proteins involved in the production of ROS [196]. Some polyphenols are also able to chelate trace metals—such as free ferrous iron (Fe^2+^) and cupric ion (Cu^2+^)—that can cause the production of aggressive free radicals [197]. The structure of the polyphenol, such as the highly conjugated systems and hydroxylation patterns of flavanols, plays an important role on its antioxidative activities [172]. Some flavonoids are particularly effective antioxidants because of the B ring hydroxyl structure, which is able to stabilize peroxyl, peroxynitrite radical, and hydroxyl by donating electrons or hydrogen [198]. 

Another mechanism through which polyphenols provide protective effects is through the modulation of autophagy [199]. Studies have found that certain polyphenols, including resveratrol, curcumin, and quercetin, have the capability of regulating autophagy, inducing programmed cell death (PCD) via the canonical (Beclin-1-dependent) and non-canonical (Beclin-1 independent) pathway [199]. This has major implications for the treatment of cancer, as polyphenols could serve as another method to control cell proliferation and induce cancerous cell autophagic cell death [200]. The enhancement of autophagy by polyphenols to clear out old, damaged, abnormal proteins and organelles may also have significant therapeutic use for neurodegenerative diseases, which are often characterized by the protein misfolding and abnormal aggregation [201].

### 4.1. Resveratrol’s Neuroprotective Effects against Parkinson’s Disease 

**Figure 3:**
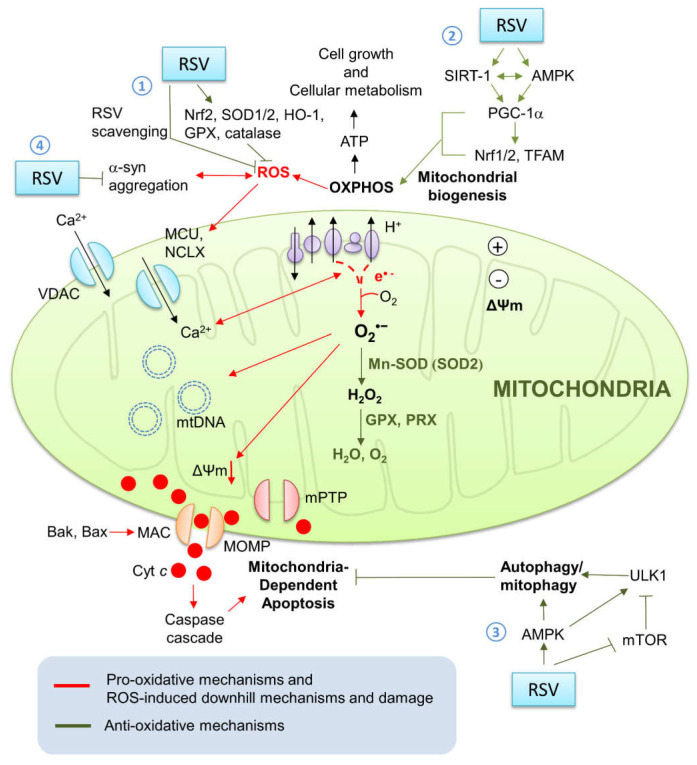
The involvement of mitochondria protection provided by resveratrol in PD pathogenesis. Normal functioning of the mitochondrial bioenergetics involves the mitochondrial OXPHOS machinery on the inner membrane transforming energy into the form of ATP in order to fuel cellular energy needs. In the process of OXPHOS, electrons leak out mainly from mitochondrial complexes I and III, producing the byproducts O_2_^•−^. The radicals may cause dyshomeostasis of Ca^2+^, damage to mtDNA, and, under overwhelming oxidative stress, even mitochondria-dependent apoptosis. Mitochondrial antioxidative mechanisms include the ROS-scavenging enzyme Mn-SOD (or SOD2), the major intracellular thiol antioxidant GPX, and the antioxidant and scavenger PRX. Resveratrol provides neuroprotective effects through: (1) scavenging cellular ROS and inducing endogenous antioxidative enzymes activities; (2) stimulating the SIRT-1-AMPK pathways and inducing the downstream PGC-1α, Nrf1/2, and TFAM to enhance mitochondrial biogenesis; (3) activating AMPK and inhibiting mTOR, which activates ULK1 and initiates autophagosome formation for autophagy/mitophagy and inhibits mitochondria-dependent apoptosis; (4) decreasing α-syn aggregation. OXPHOS, oxidative phosphorylation; ROS, reactive oxidative species; O_2_^•−^, superoxide radical; Mn-SOD (SOD2), manganese superoxide dismutase; GPX, glutathione peroxidase; SIRT-1, sirtuin 1; AMPK, 5′ adenosine monophosphate-activated protein kinase; PGC-1α, proliferator-activated receptor gamma (PPAR-γ) coactivator 1-alpha; Nrf1/2, nuclear respiratory factor 1 and 2; TFAM, mitochondrial transcription factor A; mTOR, mechanistic (or mammalian) target of rapamycin; AMPK, adenosine monophosphate-activated protein kinase, ULK1, Unc-51like kinase 1; α-syn, α-synuclein; RSV, resveratrol; SOD1/2, superoxide dismutase; HO-1, heme oxygenase-1; ΔΨm, mitochondrial membrane potential; VDAC, voltage-dependent anion-selective channel; MCU, mitochondrial calcium uniporter; NCLX, the mitochondrial Na/Li/Ca exchanger; mtDNA, mitochondrial DNA; Bak, B cell lymphoma 2 (Bcl-2) homologous antagonist killer; Bax, Bcl-2-associated X protein; MAC, mitochondrial apoptosis-induced channel; MOMP, mitochondrial outer membrane permeabilization; cyt *c*, cytochrome *c*; IMS, intermembrane space; MIM, mitochondrial inner membrane.

As mentioned previously, polyphenols are capable of exhibiting neuroprotective effects through several key mechanisms, including regulating the expression of antiapoptotic factors, inhibiting oxidant enzymes, scavenging for ROS, modulating signal transduction pathways and mitochondrial dynamics, and enhancing autophagy [201]. Below, we will delve into the properties and neuroprotective effects of a specific polyphenol, resveratrol, against PD (Figure 3). 

Resveratrol is a natural polyphenol that can be found in over 70 species of plants and their products, particularly grapes (wine), peanuts, and soy [202]. Resveratrol, or *E*-5-(4-hydroxystyryl) benzene-1,3-diol, has a stilbene structure with two phenolic rings bonded together by an ethylene bridge [202,203]. Resveratrol is a phytoalexin, meaning it is part of the active defense mechanism of plants in response to parasites, fungal infections, and other abiotic stress, such as UV light, heavy metals, respiratory inhibitors, etc. [204] Though two isometric forms of resveratrol (*cis*- and *trans*-resveratrol) exist, we will focus on the properties of *trans*-resveratrol, which has widely been associated with numerous health benefits [202,203]. 

One of the main methods through which resveratrol can offer protection against PD is by reducing oxidative stress, which has been established as a major contributor the development of PD [205]. First, resveratrol is able to scavenge for ROS and neutralize these free radicals, which can damage DNA (particularly mtDNA) and cause LDL peroxidation [206,207]. Having said that, resveratrol’s ability to scavenge for present ROS is hampered by its low bioavailability [208]. The chemical has a short biological half-life and is quickly metabolized upon entering the body, which may limit the resveratrol’s scavenging of ROS [209]. 

However, resveratrol’s ability to reduce oxidative stress goes beyond scavenging present ROS: it is also capable of inhibiting ROS production by modulating gene expression and the activity of proteins [210]. Resveratrol also reduces oxidative stress by upregulating the expression and activity of antioxidative enzymes and suppressing other ROS-generating enzymes, such as nitric oxide synthase [211,212]. For example, several studies have found that resveratrol pre-treatment can activate antioxidant enzymes SOD1 and glutathione peroxidase 1 (GPx1) [213,214,215]. Resveratrol inhibits the activity of complex III on the mitochondrial matrix side of the inner membrane, where ROS are generated, via competition with coenzyme Q [216].

Another enzyme that is involved in resveratrol’s neuroprotective effect is heme oxygenase 1 (HO-1) [217,218]. Heme oxygenase is an endogenous enzyme that provides protection against oxidative damage by degrading pro-oxidant heme into free iron, carbon monoxide, and biliverdin/bilirubin, the latter of which can further act as antioxidants [218,219]. Resveratrol selectively upregulates HO-1 expression in cultured mouse cortical neuronal cells while providing cytoprotection against free radical damage [218]. HO-1′s participation in this process was further clarified when the neuroprotective effects of resveratrol were abolished once an HO-1 activity inhibitor and protein expression inhibitor were added separately [218]. 

Resveratrol’s neuroprotective effects have also been attributed to its ability to augment autophagy, which in turn is able to prevent neuron apoptosis [217,220,221,222,223]. Lin et al., found that though rotenone (a mitochondrial complex I inhibitor capable of inducing parkinsonism symptoms) increased autophagic induction, the neurotoxin inhibited the overall autophagic flux and induced apoptosis [217,224]. When SH-SY5Y cells were treated with both rotenone and resveratrol, resveratrol was effective in preventing rotenone-induced cell death through the facilitation of autophagic induction and overall autophagic flux, respectively [217]. Once bafilomycin A1 was added to the co-treatment group, the autophagosome–lysosome fusion inhibitor prevented both the formation of acidic vesicular organelles (AVOs) and the resveratrol’s inhibition of rotenone-induced apoptosis, suggesting that resveratrol protected neurons through an autophagic manner [217]. 

Although several studies have attempted to discover the mechanism behind resveratrol’s induction of autophagy, the exact pathway remains unknown [222,225,226]. However, one of the most established and well understood pathway is resveratrol’s interaction with AMP kinase (AMPK), Unc-51 like autophagy activating kinase (ULK) 1, and mammalian target of rapamycin (mTOR) [222,225,226,227,228,229]. AMPK, a key sensor and regulator of cellular homeostasis, promotes autophagy by directly activating ULK-1 via phosphorylation of Ser 317 and 777 under nutrient insufficiency or caloric restriction (CR) [230]. On the other hand, when enough nutrients are present, mTOR prevents AMPK activation of ULK-1 by phosphorylating ULK-1 Ser 757 [230]. Resveratrol can mimic the protective effects induced by caloric restriction, inducing AMPK expression and phosphorylation [229,231]. mTOR complex I (mTORC1) regulates cell growth and promotes anabolic processes while inhibiting catabolic processes, such as autophagy [222]. Consequently, autophagy is stimulated when mTORC1 activity is inhibited, which resveratrol achieves via ATP competition by attaching to the ATP-binding site of mTOR [222]. 

As mentioned previously, mitochondrial dysfunction has been identified as a key element in the etiopathogenesis of PD. Consequently, several studies have been conducted to identify resveratrol’s impact on mitochondrial dynamics and biogenesis [231,232,233,234,235,236]. In a follow-up study concerning resveratrol’s neuroprotective effects against rotenone, Lin et al., found that rotenone induced mitochondrial fission in order to excise damaged cellular material [236]. Pre-treatment of resveratrol then partially reversed the rotenone-induced mitochondrial fragmentation via the extracellular signal-regulated kinase 1/2 (ERK1/2) pathway, enhancing mitochondrial fusion, which has been associated with the mitigation of cellular stress and a healthier mitochondrial morphology [236]. Resveratrol pre-treatment also increases the expression of mitofusin 2 and known mitochondrial biogenesis regulators peroxisome proliferator-activated receptor gamma coactivator 1-alpha (PGC-1α) and TFAM [231,235]. This effect could possibly be activated via the aforementioned AMPK pathway, as these mitochondrial markers were significantly reduced in the presence of AMPK inhibitor Compound C [231]. 

The AMPK pathway has also been noted to stimulate mitophagy through ULK-1 activation and mitochondrial biogenesis via PGC-1α-dependent transcription [231,237]. The clearance of injured mitochondria can prevent these dysfunctional organelles from releasing cytochrome c and triggering apoptosis [238]. Resveratrol pre-treatment reduces the levels of cytochrome c and activated caspase 3 in cells with MPTP or rotenone-induced mitochondrial dysfunction, reflecting a decrease in cell apoptosis [238,239]. This reduction in cell apoptosis may be able to prevent or slow the progression of PD. 

Although resveratrol’s ability to directly activate sirtuin-1 (SIRT-1) remains uncertain, reports have shown sirtuins’, particularly SIRT-1′s, neuroprotective effects against inflammation, apoptosis, and oxidative stress [240]. In a study by Albani et al., it was found that resveratrol offers neuroprotection against H_2_O_2_ or 6-hydroxydopamine (6-OHDA) via SIRT-1 activation, given that said protection was lost when SIRT-1 was downregulated [241]. Feng et al., demonstrated that SIRT-1 binding to H3K9 in the promoter region of p53 effectively inhibits p53 transcription, which has been associated with increased levels of pro-apoptotic proteins [239,242]. It has also been suggested that resveratrol could protect against PD by reducing the toxicity of α-syn aggregation via SIRT-1 activation [239,241,243]. 

Despite studies showing resveratrol being neuroprotective at the suitable dosage, there are potential adverse effects of resveratrol. Metabolites of resveratrol, such as *o*-quinone, have been associated with cytotoxic effects; studies have found them to cause hepatic and renal damage via oxidative stress and alkylation in certain situations [244,245,246,247]. Multiple studies have also shown that resveratrol exhibits biphasic concentration-dependent effects, acting as an antioxidant at low concentrations and a pro-oxidant at high concentrations [247,248,249,250,251,252,253]. For example, *o*-quinones can induce oxidative stress by depleting GSH levels and disrupting nicotinamide adenine dinucleotide phosphate oxidase (NOX) function [247,254]. Resveratrol’s pro-oxidative effects typically result in phospho-protein kinase B (PKB)/AKR mice thymoma (Akt) downregulation, cellular damage, and eventually apoptosis [247,255]. As a pro-oxidant molecule at high concentrations, resveratrol can induce DNA damage, impair multiple DNA repair pathways, and inhibit critical enzymes (e.g., DNA polymerases and ribonucleotide reductase) in the synthesis of DNA, and ultimately cause apoptosis [247,256,257,258,259]. With its ability to induce apoptosis, studies have found resveratrol to be a potential chemotherapeutic chemical by inducing apoptosis in cancerous cells (e.g., ovarian cancer cells, malignant melanoma cells, etc.) [260,261]. However, higher concentrations of resveratrol may simply induce cell death in healthy cells via its pro-apoptotic properties [247]. Ultimately, the chemical properties of resveratrol are determined by the conditions that the chemical is administered under—drug concentration, resveratrol form, time of treatment, redox state of target cell, etc. [247]. In the following section, clinical trials focused on the safety of resveratrol and its derivatives in the treatment of neurodegenerative diseases will be provided.

### 4.2. Clinical Trials of Resveratrol on Neurodegenerative Diseases

A review of the database (http://clinicaltrials.gov/, accessed on 21 May 2021) showed that over the past two decades, only seven human trials on resveratrol (BIA 6-512; trans-resveratrol) and PD have been attempted, and none had reported results (Table 2). Furthermore, all seven trials only studied the safety, optimal dosage/concentration, and pharmacokinetics of resveratrol in humans without investigating the neuroprotective benefits that they may have on humans as observed in preclinical studies. Due to the limited number of clinical studies of resveratrol on PD, a wider search of clinical trials of resveratrol on neurodegenerative diseases was conducted in order to observe the polyphenol’s safety and possible neuroprotective effects. It revealed a total of at least 16 planned, active, or completed clinical trials of resveratrol involving neurodegenerative diseases, including the seven concerning PD. The other nine clinical studies are documented in Table 3. Most of these studies are ongoing or without reported results, but two completed clinical trials on individuals with mild to moderate AD establish the safety of resveratrol in humans, particularly those with neurodegenerative conditions [262,263]. Additional clinical studies addressing the possible benefits of resveratrol on other diseases such as cancer and type 2 diabetes have established the safety of resveratrol (up to 5 g per day) for humans [264]. While this article specifically discusses the mechanisms by which resveratrol protects against PD, the polyphenol’s general antioxidative effects, ability to augment autophagy, and cytoprotective effects could be used to treat or prevent other neurodegenerative diseases.

However, even though research has consistently shown the benefits of resveratrol on neurodegenerative diseases such as AD and PD, these results have failed to be replicated in humans, most likely as a result of the resveratrol’s low bioavailability along with other pharmacokinetics [122,208,265]. Consequently, research the past few years has been focused on developing resveratrol derivatives (RVD)—such as hydroxylated, methoxylated, amidated, animated, and glycosylated derivatives—that have higher bioavailabilities, improved pharmacokinetics, and thus higher efficacy [239]. As the pharmacokinetics of resveratrol become better understood and the absorption of resveratrol/RVD becomes further enhanced, more human trials should be carried out on the efficacy of resveratrol to reduce oxidative stress, protect mitochondrial health, and ultimately provide neuroprotective effects against degenerative diseases such as PD.

## 5. Conclusions 

All in all, laboratory research has shown that resveratrol offers neuroprotective effects against PD through several key pathways. First, resveratrol reduces oxidative stress, a key part of PD’s etiopathogenesis, by scavenging for ROS, inhibiting ROS-producing enzymes, and upregulating the activity and expression of antioxidative proteins. Second, resveratrol stimulates autophagy, most notably through the AMPK pathway, enhancing the removal of damaging protein misfolding and dysfunctional organelles. Finally, resveratrol’s modulation of mitochondrial health by upregulating mitophagy and mitochondrial biogenesis prevents PD’s characteristic dopaminergic neuronal apoptosis. However, the exact mechanism through which resveratrol offers protection against PD has yet to be fully explored. 

In addition, even though resveratrol supplements have been established to be safe through clinical trials, there is not yet enough clinical evidence for its efficacy against neurodegenerative diseases. Not limited to this article’s discussion in terms of PD, once the pro-survival and beneficial mechanisms of resveratrol are more wholly understood, and more clinical trials are conducted, resveratrol can potentially be implemented in future treatment and preventive therapies not only for PD, but also for other degenerative and chronic diseases. Furthermore, resveratrol is only one of the thousands of polyphenols. This diverse group of chemicals has a wide array of possible beneficial properties, that when further investigated, could be adopted for healthcare purposes in the future. 

## Figures and Tables

**Table 1:** PD-associated genes and their functional association with mitochondria.

Symbol	Locus	Gene Name	Inheritance	Disease	Pathological Effects on the Mitochondria	Ref.
PARK1 PARK4	4q21-22	SNCA	AD	EOPD	Mutant SNCA aggregates more easily, binds to mitochondrial membranes, inhibits Complex I activity damages mitochondrial structures, and causes mitochondrial toxicity.	[138,139,140]
PARK2	6q25.2-q27	Parkin	AR	EOPD	Point mutations in parkin can inhibit its ability to interact with E2 and other protein substrates, ubiquitinate substrates, and translocate to depolarized mitochondria and induce mitophagy.	[141,142]
PARK3	2p13	Unknown	AD	Classical PD	Unconfirmed, but may be a risk factor	[143]
PARK5	4p13	UCHL1	AD	Classical PD	Mutations in UCHL1 can lead to impaired ubiquitin proteasome system (UPS), accumulation of damaged proteins, and formation of Lewy bodies.	[144,145,146,147]
PARK6	1p35-p36	PINK1	AR	EOPD	Mutations at PINK1 impair mitophagy and mitochondrial quality control by disrupting activation and recruitment of parkin to the mitochondria and the normal phosphorylation of proteins that facilitate mitophagy.	[142,148]
PARK7	1p36	DJ-1	AR	EOPD	Mutations in DJ-1 cause mitochondria damage from oxidative stress, loss of ability to prevent α-synuclein fibrillation, and increased likelihood of mitochondria depolarization and fragmentation.	[149]
PARK8	12q12	LRRK2	AD	Classical PD	Mutations in LRRK2 result in increased mitochondrial fragmentation, increased basal activity, increased susceptibility to oxidative damage, and the disruption of mitophagy.	[150,151]
PARK9	1p36	ATP13A2	AR	Kufor-Rakeb syndrome; atypical dementia with spasticity, dementia, and supranuclear glaze palsy	Mutations in ATP13A2 have been associated with reduced ATP production, increased mitochondrial fragmentation, increased ROS production, increased glycolysis (which aggravates mitochondrial dysfunction), and defective mitophagy.	[132,144,152,153,154,155,156]
PARK10	1p32	Unknown	Risk factor	Classical PD	Confirmed susceptible locus, but unknown pathology	[143]
PARK11	2q36-27	Unknown, not GIGYF2	AD	Late-onset PD	May be a risk factor, but not independently confirmed	[143]
PARK12	Xq21-q25	Unknown	Risk factor	Classical PD	Confirmed susceptible locus; may be possible risk factor; pathology unknown	[143]
PARK13	2p12	HTRA2	AD or risk factor	Classical PD	HTRA2 mutations could possibly lead to insufficient protein degradation, atypical mitochondrial morphology and function, and increased mitochondrial susceptibility to oxidative stress.	[144,157,158,159]
PARK14	22q13.1	PLA2G6	AR	Early-onset dystonia–parkinsonism	PLA2G6 participates in the regulation of Ca^2+^ within the cell. Impaired PLA2G6-dependent store-operated Ca^2+^ signaling causes autophagy dysfunction, while increased influx of Ca^2+^ into the mitochondria is associated with oxidative stress.	[144,160]
PARK15	22q12-q13	FBXO7	AR	Early-onset parkinsonian-pyramidal syndrome	Mutations in the FBXO7 gene can cause protein aggregation in the mitochondria and inhibition of mitophagy and ROS generation.	[143,161,162]
PARK16	1q32	Unknown	Risk factor	Classical PD	Confirmed susceptibility locus	[143]
PARK17	16q11.2	VPS35	AD	Classical PD	Mutations in VPS35 lead to increased mitochondrial fission/fragmentation.	[161,163]
PARK18	3q27.1	EIF4G1	AD	Classical PD	The exact mechanism of this mutation has yet to be understood.	[143,161]
PARK19	1p31.3	DNAJC6	AR	Juvenile onset, atypical PD	DNAJC6 encodes HPS40 Auxilin, but the mechanism of the mutation is not yet understood.	[164,165]
PARK20	21q22.11	SYNJ1	AR	Juvenile onset, atypical PD	SYNJ1 results in an increase in oxidative stress and change in mitochondrial morphology	[164,166]
PARK21	3q22.1	DNAJC13	AD	Late-onset PD	Mutations in DNAJC13 disrupts normal endosomal trafficking and results in α-synuclein aggregation in the lysosomes.	[164,167,168]
PARK22	7p11.2	CHCHD2	AD	Late-onset PD	Deficiency in CHCHD2 leads to reduced cytochrome c oxidase (COX) activity, decreased mitochondrial membrane potential, increased ROS production, and increased mitochondrial fragmentation.	[169,170]
PARK23	15q22	VPS13C	AR	EOPD, rapid progression	Mutations in the VPS13C gene have been associated with reduced mitochondrial membrane potential, increased mitochondrial fragmentation, and upregulated PINK1/parkin-dependent mitophagy.	[161,171]

**Table 2:** Clinical trials of resveratrol on PD.

Type of Study	Sample	Purpose	Dose	Duration	Completion Date	Main Results	Ref.
DBRCT, crossover, placebo-controlled phase I	20 healthy part.	To study resveratrol pharmacokinetics when taken together with levodopa	BIA 6-512 (trans-RSV) 25 mg, 50 mg, 100 mg dose	11 weeks	23 July 2004	Not Posted	NCT: NCT03091543
DBRCT, placebo-controlled phase I	80 healthy part.	To study the tolerability and pharmacokinetics of resveratrol and its effects on levodopa	Oral BIA 6-512 (trans-RSV) 25 mg, 50 mg, 100 mg dose	17 weeks	28 February 2005	Not Posted	NCT: NCT03091868
Single-center, open-label, RCT, two-way crossover	24 healthy part.	To study the effect of food on resveratrol pharmacokinetics	Oral BIA 6-512 400 mg dose following a breakfast (Test) or at least 8 h of fasting (Reference)	7 weeks	7 July 2005	Not Posted	NCT: NCT03095092
DBRCT, crossover, placebo-controlled phase I	40 healthy part.	To study the safety and tolerability of different doses of BIA 6-512 six times a day and to characterize the pharmacokinetics of BIA 6-512	Oral BIA 6-512 (25, 50, 100, or 150 mg dose) six times a day/4 h intervals	11 weeks	29 July 2005	Not Posted	NCT: NCT03093389
DBRCT, placebo-controlled phase I	25 part.	To compare the pharmacokinetic profile of BIA 6-512 in healthy young and old subjects	Oral BIA 6-512 200 mg every 8 h	5 weeks	2 March 2006	Not Posted	NCT: NCT03095105
Single-center, open-label, RCT, two-way crossover	39 healthy part.	To investigate the effects of BIA 6-512 at steady state on the pharmacokinetics of levodopa when administered with levodopa/benserazide with or without entacapone	Oral BIA 6-512 (25, 50, 75, and 100 mg) plus a single dose of immediate release levodopa/benserazide 200/50 mg with or without a single dose of entacapone 200 mg	7 weeks	11 July 2006	Not Posted	NCT: NCT03094156
DBRCT, crossover, placebo-controlled phase I	38 healthy part.	To investigate the effects of BIA 6-512 at steady state on the pharmacokinetics of levodopa when administered with levodopa/benserazide with or without nebicapone	Oral BIA 6-512 (25, 50, 75, and 100 mg) plus a single dose of immediate release levodopa/benserazide 200/50 mg with or without a single dose of nebicapone 150 mg	13 weeks	20 October 2006	Not Posted	NCT: NCT03097211

Abbreviation: DBRCT, double-blind randomized control trial; part., participants; h, hour; RSV, resveratrol.

**Table 3:** Clinical trials of resveratrol on non-PD neurodegenerative disease.

Type of Study	Sample	Purpose	Dose	Duration	Main Results	Completion Date	Ref.
DBRCT, placebo-controlled parallel	102 early affected Huntington disease (HD) patients	To study the therapeutic potential of RSV on the caudate volume of HD patients	RSV 40 mg twice a day	1 year	Not Posted	October 2019	NCT: NCT02336633
DBRCT, placebo-controlled Phase II	120 patients with mild to moderate dementia most likely due to AD	To study the impact on biomarkers of RSV treatment in patients with mild to moderate AD	Oral RSV 500 mg OD with dose escalation of up to 1000 mg BID	52 weeks	RSV is safe and well tolerated with nausea, weight loss, and diarrhea as side effects. No benefit on biomarkers CSF Aβ_40_ and Aβ_42_, etc. [239,263] Increased brain volume loss	March 2014	NCT: NCT01504854
DBRCT, placebo-controlled 2-period crossover, Phase II	40 Friederich ataxia (FRDA) patients	To study the efficacy of RSV as a treatment for FRDA	1 g micronized RSV or placebo twice daily for two 24 week periods	52 weeks	Recruiting	Ongoing	NCT: NCT03933163
Non-randomized, parallel assignment, open label clinical Phase I and II	27 FRDA patients (*n* = 15 will receive RSV)	To study the effects of RSV on frataxin levels in FRDA patients and to measure RSV’s effects on markers of oxidative stress, clinical measures of ataxia, and cardiac parameters	RSV 40 mg twice a day	12 weeks	Not Posted	August 2012	NCT: NCT01339884
Single center, multi-site, DBRCT, placebo-controlled Phase-3 Trial	27 mild to moderate AD patients	To investigate the efficacy of RSV in delaying the progression of AD	RSV, glucose, and malate supp. delivered in grape juice	12 months	RSV is safe and well-tolerated at low dose. No significant changes in AD Assessment Scale-cognitive subscale, Mini-Mental State Exam, etc. [239,262]	December 2010	NCT: NCT00678431
Prospective, longitudinal, mixed, analytical, experimental, double-blind, placebo-controlled study	100 amyotrophic lateral sclerosis (ALS) patients	To assess the clinical improvement of ALS patients treated with curcumin and RSV liposomed polyphenols with dutasteride	RSV 75 mg, curcumin 200 mg, and dutasteride 0.5 mg	6 months	Not Yet Recruiting	Not Yet Recruiting	NCT: NCT04654689
RCT, parallel assignment, quadruple-blind, Phase I	48 part.	To study the safety and CSF penetration of oral BDPP (grape seed polyphonic extract, RSV) in humans to assess possible benefits of BDPP to MCI	Low, moderate, and high dose of BDPP	4 months	Recruiting	Ongoing	NCT: NCT02502253
RCT, crossover assignment, open label	12 patients with hereditary spastic paraplegia (SPG5)	To study the efficacy of Xenbilox, Tahor, and RSV in decreasing oxysterols synthesis, reducing cholesterol proudction, regulating bile production, and/or providing neuroprotection	Xenbilox, Tahor, or resveratrol (80 mg for 2 months)	2 months	Not Posted	27 September 2017	NCT: NCT02314208
RCT, crossover assignment, open label, Phase I	12 patients with mild to moderate AD	To study the efficacy and safety of administering etanercept with nutritional supp. versus administering nutritional supp. alone	Nutritional supp. (curcum., luteol., theaflav., lip., acid, fish oil, quercet., resveratr.) with or without etanercept	16 weeks	Not Posted	October 2015	NCT: NCT01716637

Abbreviation: DBRCT, double-blind randomized control trial; part., participants; RSV, resveratrol; BDPP, bioactive dietary polyphenol preparation; supp., supplements; MCI, mild cognitive impairments.

## Data Availability

Not Applicable.

## Glossary

**AD**	Alzheimer’s disease	**MPTP**	1-methyl-4-phenyl-1,2,3,6-tetrahydropyridine
**ADP**	Adenosine diphosphate	**mtDNA**	Mitochondrial DNA
**AMPK**	5′-adenosine monophosphate (AMP)-activated protein kinase	**mTOR/mTORC1**	Mammalian target of rapamycin/ mammalian target of rapamycin complex I
**APAF1**	Apoptotic peptidase-activating factor 1	**nDNA**	Nucleus DNA
**ATP**	Adenosine triphosphate	**NCLX**	Na^+^/Ca^2+^ exchanger
**ATP13A2**	Atpase type13a2	**NF-** **κ** **B**	Nuclear factor kappa-light-chain-enhancer of activated B cells
**BAK**	Bcl2-antagonist/killer	**NOX**	Nicotinamide adenine dinucleotide phosphate oxidase
**BAX**	Bcl-2-associated x protein	**NRF**	Nuclear respiratory factor
**Ca^2+^**	Calcium	**6-OHDA**	6-hydroxydopamine
**CoQ**	Co-enzyme q	**OM**	Outer membrane
**CR**	Caloric restriction	**OXPHOS**	Oxidative phosphorylation
**CVD**	Cardiovascular diseases	**PD**	Parkinson’s disease
**DJ-1**	Daisuke-junko-1	**PGC**	Peroxisome proliferator-activated receptors (PPAR) γ coactivator
**ER**	Endoplasmic reticulum	**PINK1**	Phosphatase and tensin homologue (PTEN)-induced putative kinase 1
**ERK1/2**	Extracellular signal-regulated kinase 1/2	**POLG1**	Polymerase gamma 1
**ETC**	Electric transport chain	**PPAR**	Peroxisome proliferator-activated receptor
**FBXO7**	F-box only protein 7	**PRX**	Peroxiredoxin
**GPX**	Gsh peroxidase	**Redox**	Reduction-oxidization
**GR**	Glutathione reductase	**ROS**	Reactive oxygen species
**GSSG**	Glutathione disulfide	**rRNA**	Ribosomal rna
**GSH**	Glutathione	**SOD**	Superoxide dismutase
**IM**	Inner membrane	**(mt)SSB**	(Mitochondrial) single-stranded binding protein
**IMS**	Intermembrane space	**SIRT-1**	Sirtuin-1
**LRRK2**	Leucine rich repeat kinase 2	**SNpc**	Substantia nigra pars compacta
**MCU**	Mitochondrial Ca^2+^ uniporter	**TCA**	Tricarboxylic acid
**ΔΨm**	Mitochondrial membrane potential	**TFAM**	Mitochondrial transcription factor A
**MOA**	Monoamine oxidase	**TRX**	Thioredoxin
**MOMP**	Mitochondrial outer membrane permeabilization	**ULK1**	Unc-51 like autophagy activating kinase 1
**mPTP**	Mitochondrial permeability transition pore	**VPS35**	Vacuolar protein sorting 35
